# A Hybrid Deep Learning Model for Enhanced Structural Damage Detection: Integrating ResNet50, GoogLeNet, and Attention Mechanisms †

**DOI:** 10.3390/s24227249

**Published:** 2024-11-13

**Authors:** Vikash Singh, Anuj Baral, Roshan Kumar, Sudhakar Tummala, Mohammad Noori, Swati Varun Yadav, Shuai Kang, Wei Zhao

**Affiliations:** 1 Department of Instrumentation and Control Engineering, Manipal Institute of Technology, Manipal Academy of Higher Education, Udupi 576104, India; vikash.nepal@manipal.edu (V.S.); anuj.baral@learner.manipal.edu (A.B.); yadav.swati@manipal.edu (S.V.Y.); 2 Department of Electronic and Information Technology, Miami College, Henan University, Kaifeng 475004, China; henuzhao@vip.henu.edu.cn; 3 Department of Radiology, Huzhou Wuxing People’s Hospital, Huzhou Wuxing Maternity and Child Health Hospital, Huzhou 313000, China; 4 Department of Electronics and Communication Engineering, School of Engineering and Sciences, SRM University AP, Amaravati 522240, India; 5 Mechanical Engineering Department, California Polytechnic State University, San Luis Obispo, CA 93405, USA; mnoori@calpoly.edu; 6 School of Civil Engineering, University of Leeds, Leeds LS2 9JT, UK; 7 School of Civil Engineering and Architecture, Henan University, Kaifeng 475004, China; kangshuai@henu.edu.cn

**Keywords:** deep learning, ResNet-50, CNN, GoogLeNet, CBAM, damage detection

## Abstract

Quick and accurate structural damage detection is essential for maintaining the safety and integrity of infrastructure, especially following natural disasters. Traditional methods of damage assessment, which rely on manual inspections, can be labor-intensive and subject to human error. This paper introduces a hybrid deep learning model that combines the capabilities of ResNet50 and GoogLeNet, further enhanced by a convolutional block attention module (CBAM), proposed to improve both the accuracy and performance in detecting structural damage. For training purposes, a diverse dataset of images depicting both structural damage cases and undamaged cases was used. To further enhance the robustness, data augmentation techniques were also employed. In this research, precision, recall, F1-score, and accuracy were employed to evaluate the effectiveness of the introduced hybrid deep learning model. Our findings indicate that the hybrid deep neural network introduced in this study significantly outperformed standalone architectures such as ResNet50 and GoogLeNet, making it a highly effective solution for applications in disaster response and infrastructure maintenance.

## 1. Introduction

**Figure 1:**
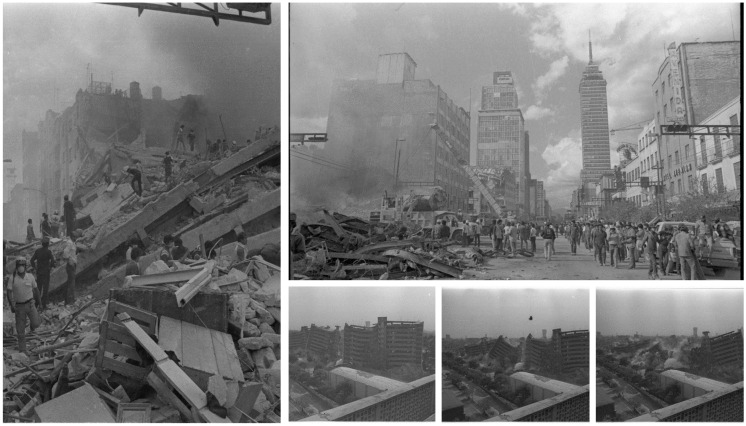
Images of the 1985 Mexico Earthquake, from Mexico City (https://commons.wikimedia.org/wiki/File:Album_de_imagenes_del_terremoto_de_1985_UsoLibre.png, (accessed on 2 November 2024)).

When it comes to disaster management, timely and precise structural damage assessment is vital for effective emergency response and recovery efforts. It is crucial for maintaining the safety and integrity of buildings and infrastructure in everyday maintenance, as well as in the aftermath of disasters. Images of the 1985 Mexico earthquake, from Mexico City, are shown in Figure 1 (https://commons.wikimedia.org/wiki/File:Album_de_imagenes_del_terremoto_de_1985_UsoLibre.png, (accessed on 2 November 2024)).

However, the traditional methods of evaluation mostly rely on human inspection and have been shown to be labor-intensive, often hindering the ability to make critical decisions in a timely manner [1]. However, the emergence of deep learning technology has revolutionized image classification, offering automated solutions for tasks that formerly necessitated substantial human involvement [2].

This study offers a model designed to evaluate not only visible cracks but also other critical signs of structural damage. These include partial displacements, which may indicate foundational shifts; deformations, which might indicate excessive stress; and surface abnormalities and fractures, which may eventually cause a structure to weaken over time. This more comprehensive framework for assessment is crucial for routine maintenance and monitoring, as well as for post-disaster scenarios. It allows for early action that ensures structural resilience and stops additional damage [3,4].

Prior research has highlighted the capability of deep learning for structural damage detection. For instance, Lee et al. utilized convolutional neural networks (CNNs) to detect cracks in building facades, achieving an accuracy of 95.3% [5]. In the context of infrastructure inspection, Yang et al. employed deep learning for crack detection in images captured by unmanned aerial vehicles (UAVs), reporting an average precision of 93.7% and a recall of 91.2% [6]. These studies, along with others, showcase the growing promise of deep learning, to enhance and automate the accuracy of damage assessment processes [7,8].

Furthermore, research has shown the applicability of deep learning across different types of structures and damage indicators. Yuqing and Khalid applied the VGG16 model for structural damage recognition, achieving 90% accuracy [9]. Similarly, Cao et al. used VGG16 to detect cracks in the gusset plate welded joints of steel bridges, achieving an accuracy of 94% [10]. Xiuying leveraged a ResNet101-based image segmentation model for concrete crack detection, achieving 94.52% precision and 95.25% recall accuracy, underscoring the importance of deep residual networks in such tasks [11].

Zheng et al. used YOLOv3 and RetinaNet pre-trained on the COCO dataset in surface crack detection to effectively identify cracks [12]. Additionally, Guo et al. applied the YOLOv5 model to pavement crack detection, achieving 88.1% accuracy, showcasing its utility in infrastructure maintenance [13]. Kong and Li proposed a vision-based method for detecting metal fatigue cracks using video feature tracking, which is crucial for ensuring the safety and longevity of steel structures [14]. Furthermore, Wilson and Diogo demonstrated an approach with the VGG16 model, achieving 92.27% accuracy with a dataset of fewer than 3500 images [15]. Wan, Shuai et al. discussed the integration of knowledge-driven and data-driven techniques in SHM, presenting a framework that enhances monitoring accuracy by combining the strengths of both approaches [16]. Furthermore, Khan, Imdad Ullah et al. explored the challenges of anomaly detection for long-term SHM data with deep-learning and rule-based classification methods. They examined issues such as ambiguous data categorization, information loss in time-series conversion, and thresholding requirements, contributing to the development of more reliable anomaly detection in SHM systems [17].

Our research introduces a novel CNN model, designed for the task of building damage detection. By integrating a dual attention mechanism and drawing inspiration from established architectures like ResNet and GoogLeNet, our model achieved a remarkable accuracy of 98.6%. This advancement highlights the significance of customizing deep learning solutions for damage assessment applications. The model’s exceptional performance, evidenced by its high precision (98.2%), recall (98.8%), and F1-score (98.5%), further demonstrated the model’s effectiveness, ultimately contributing to the development of safer and more resilient infrastructure [18,19].

Our approach also uses transfer learning techniques, which significantly enhance the models’ performance and efficiency. Our models are better equipped to handle targeted tasks by applying knowledge acquired from large scale datasets to our specific scenario [20,21]. The deep residual learning features of ResNet-50 and the inception modules of GoogLeNet (both pre-trained on the ImageNet dataset) enable our models to extract features from damaged structure images, for improved classification accuracy and reliability. Our ultimate objective (i.e., our future goal) is to deploy this model alongside drone technology, enabling drones to capture real-time images of buildings in the aftermath of a disaster. Equipped with GPS tagging, the captured images could be transmitted back to a base station, where the model could rapidly assess damage levels and locations. This approach would allow disaster response teams to gauge the extent and exact location of structural damage, enabling them to allocate resources and personnel more efficiently. By first developing a robust, accurate classification algorithm and then integrating it into a real-time system, we aim to create a tool that prioritizes critical areas, ultimately saving time, resources, and potentially lives during rescue operations.

## 2. System Model

In this section, we briefly explore CNNs, Resnet50, GoogLeNet, and the proposed model.

### 2.1. Convolutional Neural Networks (CNNs)

**Figure 2:**
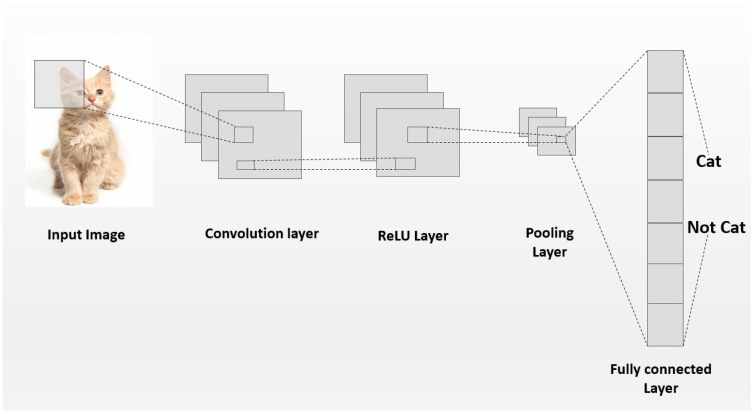
A Basic CNN architecture, illustrating the convolutional, pooling, and fully connected layers used to extract spatial features for structural damage detection.

CNNs are a class of deep learning models tailored for handling data with a structured grid-like arrangement, such as in the case of images. Unlike traditional neural networks, CNNs are characterized by their use of convolutional layers. Several filters are applied across the input data to automatically and adaptively learn the features in a spatially hierarchical manner [22]. The CNN architecture is shown in Figure 2, and the fundamental building block of a CNN is the convolutional layer, which performs the convolution operation, given in (1).

(1) $$F(i,j)=\sum_{m}\sum_{n}X(i+m,j+n)W(m,n)$$

where X is the input image or feature map; W is the filter or kernel; m,n are indices that represent the spatial dimensions of the filter (or kernel); F is the feature map output after the convolution; and i,j are the indices across the dimensions of the feature map.

After the convolution operation, a non-linear activation function, such as a rectified linear unit (ReLU), is applied. This introduces non-linearity, enabling the model to capture more complex patterns [23]. Furthermore, pooling layers are typically used to gradually decrease the spatial dimensions of the feature maps, which helps to lower the number of features and the computational overhead, while preserving the most significant features [24]. Finally, fully connected layers combine these extracted features for classification and use a function (e.g., the softmax function) to output probabilities [25].

### 2.2. ResNet50

**Figure 3:**
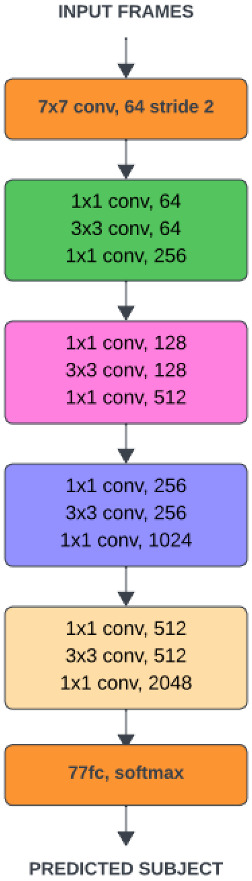
Basic architecture of ResNet50.

ResNet50’s deep architecture is shown in Figure 3. It is widely recognized for its ability to train very deep networks, while avoiding the vanishing gradient problem through the use of residual learning. ResNet50 has residual blocks, an innovation that enables the network to learn identity mappings.

ResNet50 is a well-established deep convolutional neural network, recognized for its capacity to effectively train very deep architectures. This is achieved by addressing the vanishing gradient problem through residual learning. The core innovation in ResNet50 lies in its residual blocks, which facilitate the learning of identity mappings. Mathematically, a residual block is represented in (2).

(2) $$\begin{array}{c} y=F(x,\left\{W_{i}\right\})+x \end{array}$$

In this expression, *x* is the input to the block, and $F(x,\left\{W_{i}\right\})$ denotes the residual function. This function typically comprises convolutional layers, batch normalization, and ReLU activations. The set $\left\{W_{i}\right\}$ represents the weights associated with these layers, which are adjusted during training.

The addition operation in the equation enables the network to learn a residual mapping, which proves to be easier to optimize than learning a direct mapping [26]. ResNet50 is structured with multiple residual blocks, each designed to effectively capture and propagate complex features across different layers [27].

ResNet50’s deep architecture is particularly well-suited for tasks that require detailed feature extraction, such as detecting structural damage, where subtle indicators like cracks need to be identified. However, despite these strengths, the model may encounter challenges in efficiently processing multi-scale features. This constraint has driven research toward exploring alternative architectures such as GoogLeNet [28], which provide innovative strategies for addressing multi-scale feature representation.

### 2.3. GoogLeNet

**Figure 4:**
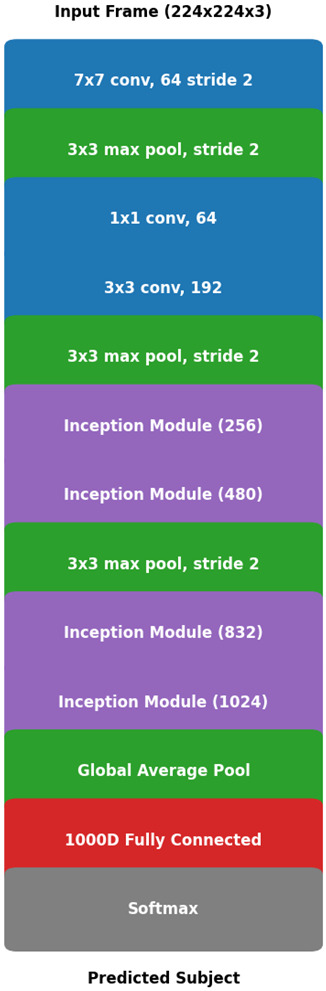
GoogLeNet architecture, featuring inception modules that process multiple filter sizes in parallel to capture varied structural damage features within images.

The GoogLeNet architecture is shown in Figure 4. This model is also referred to as Inception-v1 and brought several groundbreaking features that distinguished it from earlier convolutional neural networks (CNNs). These features, notably the inception modules, enhanced its capability to handle multi-scale data processing. The inception module applies several convolutional filters of sizes 1 × 1, 3 × 3, and 5 × 5 in parallel, followed by a max-pooling operation, as illustrated in Figure 4. This design allows the network to capture both fine and coarse features within a single layer, enhancing its ability to process diverse scales of information. The output of the inception module is expressed in (3).

(3) $$\mathrm{Inception}\;\mathrm{Output}=[f_{1\times 1}(x),f_{3\times 3}(x),f_{5\times 5}(x),\mathrm{MaxPool}(x)]$$

where $f_{1\times 1}$, $f_{3\times 3}$, and $f_{5\times 5}$ are convolutional operations with kernel sizes of 1×1, 3×3, and 5×5, respectively, applied to the input *x*. The MaxPool(x) operation refers to a max pooling layer, which extracts the maximum value from a specified region, thereby reducing the spatial dimensions. The final output is obtained by concatenating the feature maps generated by each of these operations.

GoogLeNet’s architecture is computationally efficient, while maintaining high accuracy, which is particularly advantageous for tasks involving input data with features at different scales [28]. The success of this design influenced the development of later models, such as Inception v3 and Inception v4, which further refined multi-scale processing [29]. This multi-scale processing capability of GoogLeNet is especially useful in detecting structural damage, where features such as cracks may vary in size and location across different regions of an image [30]. Despite its strengths, GoogLeNet, like ResNet50, may not always emphasize the most relevant features. Therefore, to enhance performance, attention mechanisms have been incorporated into newer models to ensure that the network focuses on the most critical aspects of the input data.

## 3. Proposed Model

The proposed model integrates the strengths of two well-established deep learning architectures—ResNet50 [26] and GoogLeNet [28]—to create a robust and flexible framework for detecting structural damage using images. By combining these architectures with an advanced attention mechanism, namely a convolutional block attention module (CBAM), the model is designed to focus on the most critical features within the image, thereby enhancing its overall performance in identifying structural anomalies [31].

**Figure 5:**
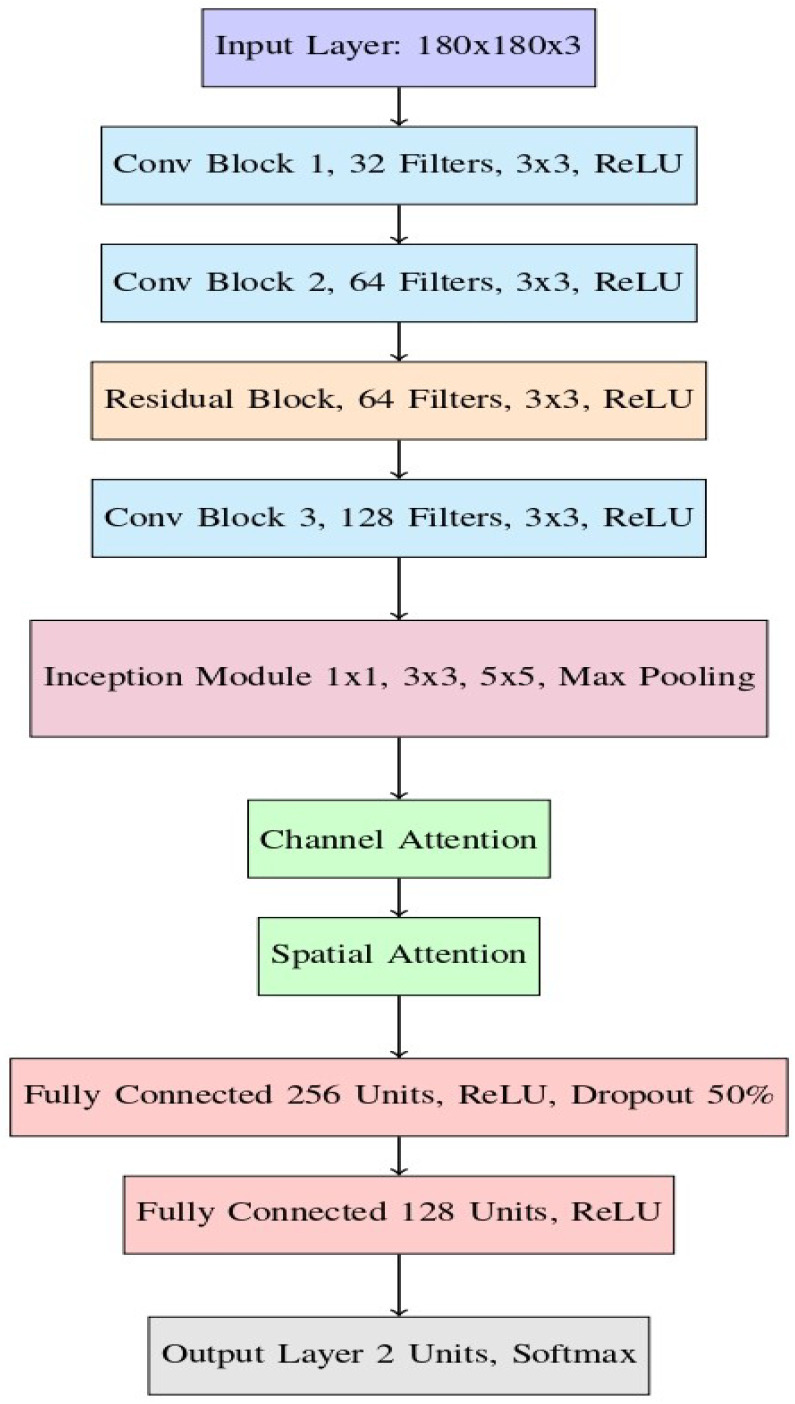
Proposed hybrid model layout, integrating ResNet50, GoogLeNet, and attention mechanisms to improve the feature extraction and classification accuracy for structural damage detection.

The comprehensive layout of the presented model is depicted in Figure 5. It is composed of several key components, each playing an important role in the feature extraction and classification. These components include convolutional blocks, a residual block, an inception module, and an attention mechanism, followed by fully connected layers that output the final classification decision.

The model accepts images of 180 × 180 × 3 dimensions, suitable for capturing the necessary details while maintaining computational efficiency, as depicted in Figure 5.

### 3.1. Convolutional Blocks

**Figure 6:**
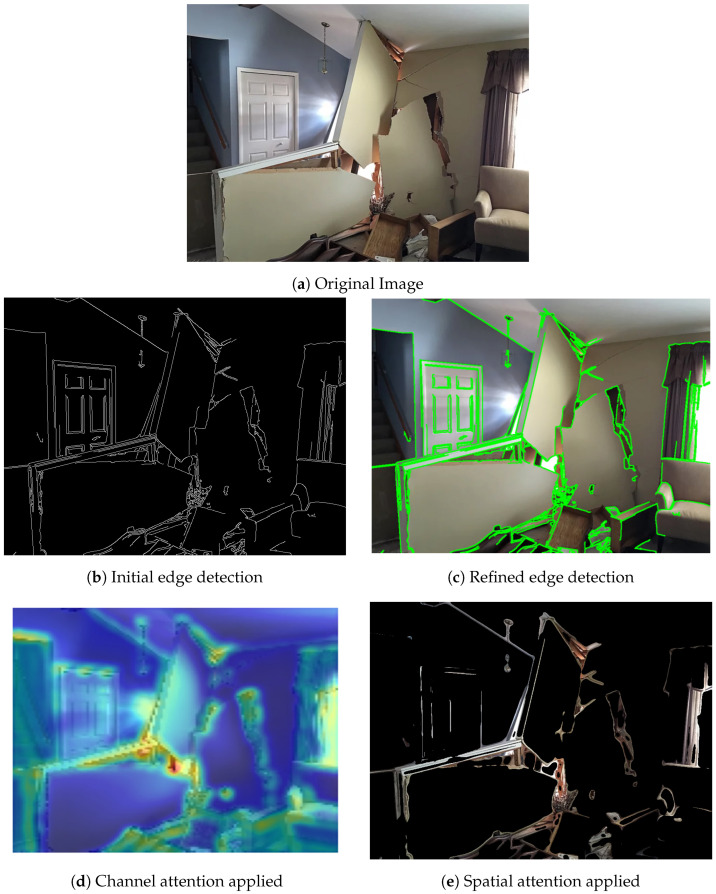
Transformation stages of the input image through convolutional layers, highlighting the initial edge detection and refinement with the convolutional block attention module (CBAM), (**a**) original image (**b**) initial edge detection, (**c**) refined edge detection, (**d**) channel attention applied, and (**e**) spatial attention applied.

The feature extraction process begins with a series of three convolutional blocks, each designed to progressively capture more complex and abstract features from the input images. The purpose of these convolutional blocks is to identify low-level characteristics, including edges, textures, and patterns Each convolutional block contains multiple filters, with each filter learning to detect unique features from the input image. Figure 6 illustrates how these filters progressively transform the input image, highlighting the distinct features captured at each stage.

- **Conv Block 1:** The initial convolutional block uses 32 filters of size 3 × 3 to process the input image. This is followed by a ReLU activation function, which introduces non-linearity into the model to learn the more complex patterns. This block primarily detects fundamental features like edges and simple textures, as illustrated in Figure 6b, which serve as a foundation for deeper layers in the network.
- **Conv Block 2:** The output from the first block is passed to the second convolutional block, where the filter size is 3 × 3 with 64 filters. Similarly to the first block, a ReLU activation function is applied. This block builds upon the basic features captured in the first block, allowing the model to detect more complex structures and patterns within the images, such as corners and intricate textures. The refined edge detection result at this stage is shown in Figure 6c.
- **Conv Block 3:** The third convolutional block further increases the complexity of the feature extraction by applying 128 filters of size 3 × 3, followed by ReLU activation. This block is crucial for capturing high-level features that are directly related to structural damage, such as cracks, fractures, and deformations in the building structures.

### 3.2. Residual Block

After the convolutional blocks, the architecture incorporates a residual block inspired by ResNet50. The residual block is used to avoid the vanishing gradients problem arising in deep neural networks by using skip connections, such that the model can learn residual mappings. These mappings enable the network to retain and reuse learned features across layers, thereby improving the model’s capacity to recognize patterns, without any loss in performance. Mathematically, a residual block is represented in (2). This formulation allows the network to learn the difference between the input and the desired output, rather than attempting to learn the entire transformation all at once.

### 3.3. Inception Module

The output from the residual block is fed into an inception module, which is a critical component borrowed from GoogLeNet. The inception module is designed to perform multi-scale feature extraction by applying multiple filters of different sizes (e.g., 1 × 1, 3 × 3, and 5 × 5) in parallel, followed by max pooling. This architecture allows the model to capture features at various scales, making it particularly effective in identifying structural damages of different sizes and shapes within the same image. The inception module is mathematically represented in (3), where the outputs from parallel filters are concatenated to form a comprehensive feature map that captures the multi-scale features necessary for accurate structural damage detection.

### 3.4. Attention Mechanism (CBAM)

**Figure 7:**
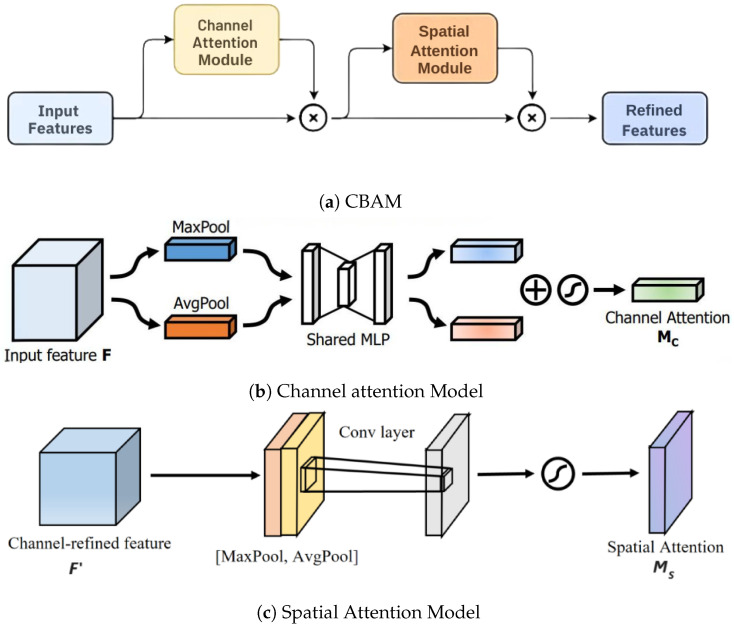
Convolutional block attention module (CBAM) components, consisting of channel attention and spatial attention models, (**a**) CBAM, (**b**) channel attention model, and (**c**) spatial attention model.

Following the inception module, a convolutional block attention module (CBAM) is applied to enhance the feature maps by emphasizing the most important elements of the image. As depicted in Figure 7a, the CBAM comprises two crucial components: channel attention and spatial attention, which work together to refine the focus on relevant features.

- **Channel Attention:** This component enhances the model’s ability to focus on the most informative feature channels within the feature map. The channel attention mechanism operates by applying both global average pooling and global max pooling across the spatial dimensions of the input feature map F. This generates two separate context descriptors, which are then processed through a shared multi-layer perceptron (MLP) to capture channel-wise dependencies and create the final channel attention map. The steps of this process are detailed below.
  First, we apply global average pooling and global max pooling operations to F, generating two channel-wise statistics, $F_{\mathrm{avg}}$ and $F_{\max}$:
  (4) $$F_{\mathrm{avg}}=\mathrm{AvgPool}(F)$$
  (5) $$F_{\max}=\mathrm{MaxPool}(F)$$
  where $F_{\mathrm{avg}}$ and $F_{\max}$ represent the globally pooled feature vectors for each channel.
  Next, these descriptors are passed through a shared MLP, which consists of two fully connected layers. The shared MLP generates intermediate feature representations for both average-pooled and max-pooled inputs. The two MLP outputs are then summed element-wise to produce the final channel attention map:
  (6) $$M_{\mathrm{channel}}(F)=\sigma (\mathrm{MLP}(F_{\mathrm{avg}})+\mathrm{MLP}(F_{\max}))$$
  where σ is the sigmoid activation function, which normalizes the channel attention map values to the range [0,1].
  To further expand on the shared MLP, we can represent it as a sequence of two fully connected layers. If the intermediate representation has *d* dimensions, we can define the MLP as
  (7) $$\mathrm{MLP}(F)=W_{2}\;\delta (W_{1}\;F+b_{1})+b_{2}$$
  where
  $W_{1}$ and $W_{2}$ are weight matrices for the first and second fully connected layers, respectively,
  $b_{1}$ and $b_{2}$ are the bias terms for each layer,
  δ is the ReLU activation function, which introduces non-linearity after the first fully connected layer.
  Finally, the channel attention map $M_{\mathrm{channel}}F$ is used to reweight the original feature map F by element-wise multiplication:
  (8) $$F_{\mathrm{refined}}=M_{\mathrm{channel}}(F)⊙F$$
  where ⊙ denotes element-wise multiplication, and $F_{\mathrm{refined}}$ is the enhanced feature map that emphasizes the most important channels for the task. The entire process highlights the channel attention mechanism’s ability to selectively enhance meaningful feature channels, as illustrated in Figure 6d.
- **Spatial Attention:** Following the channel attention, spatial attention is applied to focus on the most critical spatial locations within the feature map. This component applies average and max pooling operations, and then a convolutional layer, in order to generate the spatial attention map, as in Figure 7c. The spatial attention mechanism can be described by applying convolutional operations to the max-pooled and average-pooled feature maps, focusing on emphasizing the most significant spatial regions within the feature map. This results in the generation of a spatial attention map.
  (9) $$M_{\mathrm{spatial}}(F)=\sigma (f^{7\times 7}(\left[\mathrm{AvgPool}(F);\mathrm{MaxPool}(F)\right]))$$
  where $M_{\mathrm{spatial}}(F)$ refines the model’s focus on crucial spatial regions within the feature maps, thereby improving the accuracy of structural damage detection. The operations AvgPool(F) and MaxPool(F) correspond to global average pooling and global max pooling, respectively, while $f^{7\times 7}$ represents a convolutional operation using a 7×7 filter. The sigmoid activation function, denoted by σ, is applied to normalize the resulting output within the range of [0, 1]. The impact of spatial attention is illustrated in Figure 6e.
- **Sequential Dual Attention Application:** The dual attention mechanism operates sequentially within both architectures. Each feature map is first processed by the channel attention module to prioritize significant channels and then by the spatial attention module to refine the focus on relevant regions within the image. This two-step attention approach enables the model to capture intricate damage patterns by focusing both on the most meaningful channels and specific spatial locations within each feature map.
- **Technical Feasibility:** The CBAM is a lightweight module and, when applied after each main block in ResNet50 and GoogLeNet, adds minimal computational overhead. This design choice allows the model to leverage dual attention without a substantial increase in processing time or resource requirements, maintaining the efficiency of ResNet50 and GoogLeNet. The final architecture thus benefits from enriched feature representation, with an enhanced capacity for damage localization and detection.

### 3.5. Fully Connected Layers and Output

After the attention mechanisms, the network transitions to the fully connected layers used for the final stages of feature extraction and classification:

- **Fully Connected Layer 1:** This layer consists of 256 units utilizing the ReLU activation function, with a dropout rate of 50% incorporated to mitigate overfitting during training. It plays a crucial role in combining the features extracted by the previous layers and preparing them for the final classification.
- **Fully Connected Layer 2:** This layer contains 128 units with ReLU activation. It further reduces the dimensionality of the feature maps, ensuring that only the most relevant features are passed on to the output layer.
- **Output Layer:** The final layer of the network is a softmax output layer with two units, corresponding to a binary classification task of determining whether a structure is “damaged” or “undamaged”. The softmax function ensures that the outputs are interpretable as probabilities, summing to 1 across the two classes.

## 4. Workflow of the System Model

**Figure 8:**
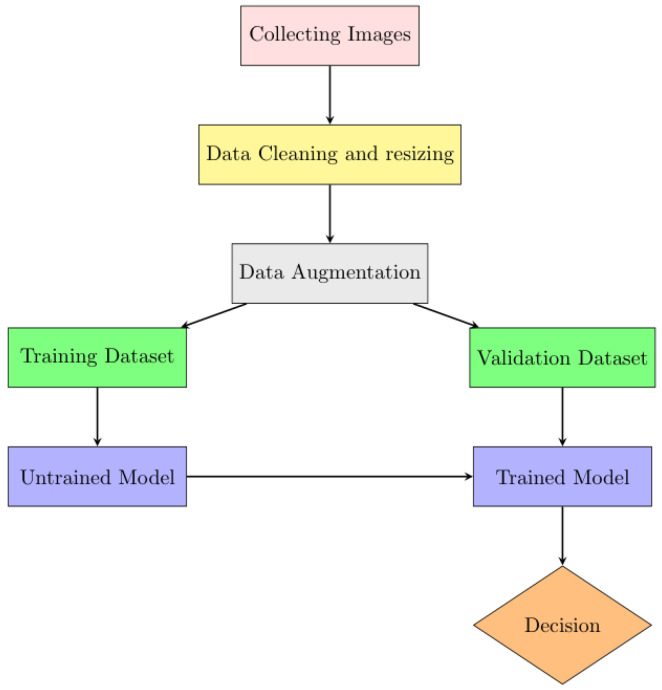
Workflow of the system model.

The workflow of the proposed system model is visually summarized in Figure 8. The process begins with the careful assembly of a well-labeled dataset. Once data collection is complete, the images are resized to ensure compatibility both at the time of training and validation phases. Finally, the model was validated using random inputs to assess its performance. The function of each block is thoroughly discussed in the following subsection.

### 4.1. Data Collection and Filtering

**Figure 9:**
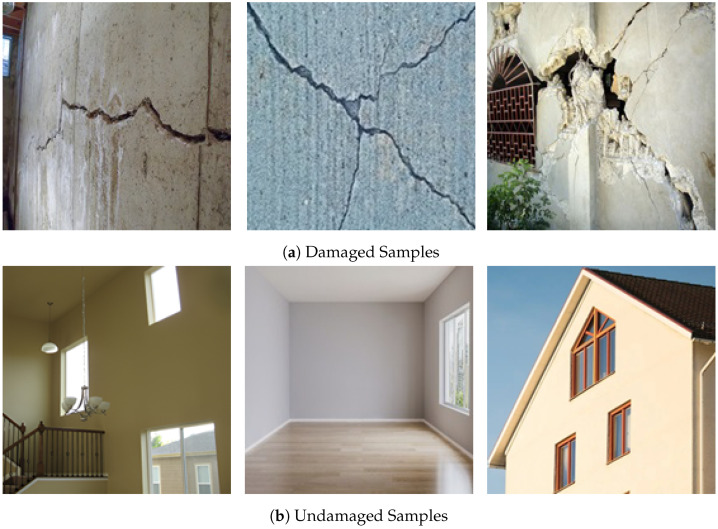
Samples of (**a**) damaged structures and (**b**) undamaged structures.

In this study, data were collected from diverse sources to ensure a comprehensive representation of both damaged and non-damaged structures. Some sample images from the datasets are shown in Figure 9. The key sources for data collection were as follows:

- **Bing Images:** Bing’s search engine was utilized to retrieve images using targeted keywords like “earthquake structural damage”, “structural cracks”, and “building deformation”. The search was broadened with additional terms such as “flood-damaged buildings”, “tornado-damaged structures”, and “infrastructure failure”.
- **Kaggle Dataset:** A specialized dataset from Kaggle was employed (https://www.kaggle.com/datasets/arnavr10880/concrete-crack-images-for-classification/data (accessed on 15 January 2024)), focusing on images of concrete slabs, both cracked and uncracked, relevant for early damage detection.
- **Public Repositories:** Additional relevant images were sourced from public datasets, including the “Structural-Damage Image Captioning Dataset” repository (https://jstagedata.jst.go.jp/articles/dataset/Structural-Damage_Image_Captioning_Dataset/24736914 (accessed on 15 January 2024)), known for its well-curated collection categorized by damage type.

#### 4.1.1. Outlier Detection and Removal

Maintaining the dataset’s integrity involved a stringent process of outlier detection and removal:

- **Visual Inspection:** A manual review was conducted to eliminate irrelevant, low-quality, or mislabeled images. Special attention was given to ensuring that undamaged structure images did not contain features resembling cracks.
- **Statistical Analysis:** Statistical analysis of pixel intensity distributions was performed. Images with significant deviations were flagged as potential outliers, and further feature-based analysis was used to detect anomalies, which were then removed to maintain data consistency.

#### 4.1.2. Data Classification and Standardization

Following the data cleaning process, all images were resized uniformly to 180 × 180 pixels and converted to RGB format to ensure consistency across the dataset, facilitating uniform analysis and model training. The dataset was then categorized into two groups:

- **Damaged:** This category contains 1600 images that exhibit various levels of structural damage, such as cracks and deformations, as illustrated in Figure 9a.
- **Undamaged:** This group consists of images depicting structures that are largely intact, with little to no visible damage, as shown in Figure 9b.

### 4.2. Data Augmentation

**Figure 10:**
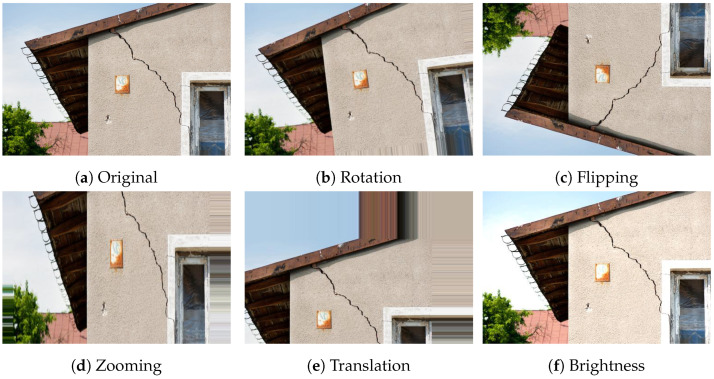
Images after data augmentation operations: (**a**) original, (**b**) rotation, (**c**) flipping, (**d**) zooming, (**e**) translation, and (**f**) brightness.

To enhance the robustness and generalization of the neural networks used, several data augmentation methods were applied. These methods are especially beneficial when working with limited datasets, as they expand the training set by generating modified versions of the original images. Sample augmented images are shown in Figure 10, where Figure 10a represents the original image. The following augmentation techniques were applied to the original image:

- **Rotation:** Images were rotated randomly within a range of −20° to +20° to help the model become invariant to the orientation of structural damage, a sample rotated image is shown in Figure 10b.
- **Horizontal and Vertical Flipping:** Both horizontal and vertical flips were applied to ensure that the model learned to recognize damage patterns irrespective of their orientation, a sample rotated image is shown in Figure 10c.
- **Zooming:** Zooming operations were applied randomly within a range of 0.8 to 1.2 times the original size, allowing the model to handle scale variation in damage features, a sample rotated image is shown in Figure 10d.
- **Translation:** Images were translated horizontally and vertically by up to 10% to enable the model to detect damage appearing in different image locations, a sample rotated image is shown in Figure 10e.
- **Brightness Adjustment:** In image-based structural damage detection, variations in illumination can significantly affect the model accuracy, as differences in lighting alter the contrast and visibility of critical features like cracks and deformations [32]. Rather than relying on preprocessing techniques like histogram equalization or normalization to adjust illumination, our approach introduces controlled brightness variation directly through data augmentation. Specifically, the brightness of images was adjusted within a range of 0.8 to 1.2, which exposed the model to a spectrum of lighting conditions during training. This augmentation strategy helps the model generalize to real-world scenarios where lighting can vary, thereby enhancing the robustness in detecting structural damage across diverse environments without the need for additional preprocessing steps. A sample of a brightness-improved image is shown in Figure 10f.

These augmentation techniques were applied during training, significantly increasing the dataset, and thereby ensuring a data balance and exposing the model to a diverse range of training examples.

### 4.3. Data Splitting

Post-augmentation, the dataset was split into training, validation, and testing subsets, with 70% allocated for training, 15% for validation, and 15% for testing. This split ensured that the model could be trained, validated, and tested on distinct portions of the data, reducing the risk of overfitting and enhancing its generalization capabilities.

### 4.4. Training and Validation

The training and validation process was divided into two stages. Initially, pre-trained models were used to assess the performance, followed by the evaluation of a custom model to address any limitations observed. In the first stage, ResNet50 and GoogLeNet were selected due to their proven success in image classification tasks. These models were fine-tuned on the dataset with the predefined splits. Both models were trained over 30 epochs using w stochastic gradient descent (SGD) optimizer, with a momentum parameter of 0.9. The learning rate was set to 0.001 and was progressively reduced by a factor of 0.1 every 10 epochs. A batch size of 32 was chosen to ensure a balance between computational efficiency and model performance. Additionally, early stopping and a dropout rate of 0.2 were applied to prevent overfitting, halting training if no improvement was observed over five consecutive epochs. This approach ensured that the models retained their ability to generalize to unseen data [33].

This careful hyperparameter tuning allowed the models to converge more smoothly, as reflected in the gradual improvement in the loss curves, without significant spikes.

To enhance the performance and minimize the risk of overfitting, a custom CNN model was developed and trained on the same dataset. This model followed the same training procedure, using the same SGD optimizer, dropout, and early stopping strategy, and the custom model also benefited from a gradual reduction in the learning rate and smooth convergence, as reflected in the loss curves. Training in this manner was not biased toward a particular comparison, as all models were evaluated under similar conditions.

## 5. Results and Analysis

In this section, both the pre-trained models and the custom CNN were evaluated based on accuracy, precision, recall, and F1-score. The area under the receiver operating characteristic curve (AUC-ROC) provided additional understanding of the models’ classification capabilities [34]. After the training process, a confusion matrix was produced for each model to analyze their performance in more detail. A confusion matrix was utilized to delve more deeply into our model’s damage detection capabilities, offering insights into its accuracy, precision, recall, and error distribution.

The system used for the computational analysis was an Intel Core i7-9700K processor with eight cores at a base clock speed of 3.6 GHz, along with 32 GB of DDR4 RAM and an NVIDIA RTX 2060 GPU, which had 6 GB of GDDR6 VRAM for graphics processing.

(10) $$\begin{array}{c} \mathrm{Precision}=\frac{\mathrm{TP}}{\mathrm{TP}+\mathrm{FP}} \end{array}$$

where TP is true positives and FP is false positives

(11) $$\begin{array}{c} \mathrm{Recall}=\frac{\mathrm{TP}}{\mathrm{TP}+\mathrm{FN}} \end{array}$$

where FN is false negatives.

(12) $$\begin{array}{c} \mathrm{F1-Score}=2\times \frac{\mathrm{Precision}\times \mathrm{Recall}}{\mathrm{Precision}+\mathrm{Recall}} \end{array}$$

(13) $$\begin{array}{c} \mathrm{Accuracy}=\frac{\mathrm{TP}+\mathrm{TN}}{\mathrm{TP}+\mathrm{TN}+\mathrm{FP}+\mathrm{FN}} \end{array}$$

where TN is true negatives and FP is false positives.

The models’ performance was comprehensively assessed using a separate test set that was not involved in the training or validation phases. This assessment aimed to provide an accurate evaluation of each model’s capability to generalize to new, unseen data.

### 5.1. Performance of Pre-Trained Models

ResNet50 and GoogLeNet were the first models we tested on the damage detection task. These models were fine-tuned on our dataset and yielded strong performances, although with some notable differences.

#### 5.1.1. ResNet50 Performance

**Figure 11:**
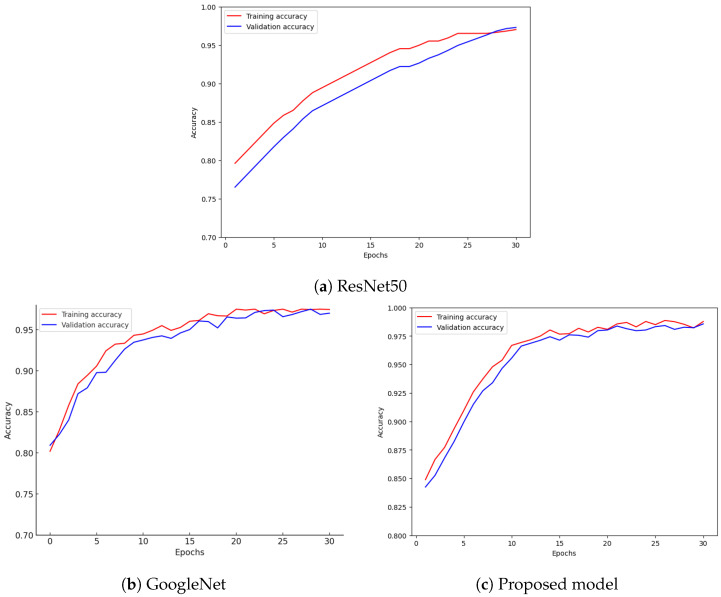
Training and validation accuracy for (**a**) ResNet50, (**b**) GoogLeNet, and (**c**) proposed model.

The ResNet50 model achieved an accuracy of 97.2%, with a precision of 97.1%, recall of 96.9%, and an F1-score of 97.0%. These metrics demonstrate the model’s ability to effectively detect damaged structures, while maintaining a good trade-off between precision and recall. The accuracy graph for ResNet50 (Figure 11a) shows the consistent improvement and strong alignment between the training and validation curves. However, there was a minor risk of overfitting, as the training accuracy remained higher than that of the validation set, suggesting the model learned patterns from the training data better than from the unseen validation data.

#### 5.1.2. GoogLeNet Performance

GoogLeNet demonstrated a slightly higher accuracy of 97.5%, with a precision of 97.5%, recall of 97.3%, and an F1-score of 97.4%. These results suggest that GoogLeNet’s multi-scale feature extraction capability allowed it to perform exceptionally well in detecting a wide range of structural damages. The accuracy graph for GoogLeNet (Figure 11b) indicates smooth and consistent learning, with both training and validation accuracies reaching high levels and showing minimal signs of overfitting.

Comparing the two models, GoogLeNet performed slightly better than ResNet50 in accuracy and overall metrics. However, both models demonstrated strong generalization capabilities, as evidenced by the close alignment of their training and validation curves. The confusion matrices further illustrate the effectiveness of both models in accurately classifying damaged and undamaged structures.

#### 5.1.3. Performance of Proposed Model

The performance of our proposed custom CNN model addressed the limitations observed in the pre-trained models, enhancing the accuracy and reliability of structural damage detection. This custom model integrates advanced architectural elements, including residual connections, inception modules, and a convolutional block attention module (CBAM), which together improve its ability to detect damage accurately across varied scenarios. The use of residual connections enables efficient feature propagation and deeper network layers, without vanishing gradients, while the inception modules capture features at multiple scales, essential for recognizing diverse types of structural damage. The CBAM further refines the model by applying channel and spatial attention, selectively focusing on critical features in each image, which enhances the model’s sensitivity to subtle damage indicators.

**Figure 12:**
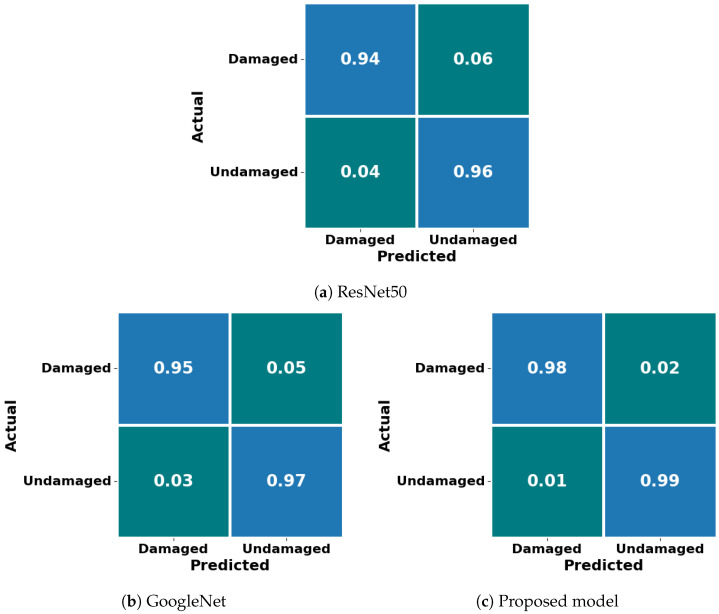
Confusion matrix for (**a**) ResNet50, (**b**) GoogLeNet, and (**c**) proposed model.

The proposed model achieved an accuracy of 98.6%, with a precision of 98.2% and a recall of 98.8%, leading to an F1-score of 98.5%. These metrics highlight the model’s strong capability to accurately detect damaged structures, while minimizing false positives and negatives. Figure 12a–c present the confusion matrices for ResNet50, GoogLeNet, and the proposed model, respectively. The proposed model’s confusion matrix demonstrates a higher number of true positives and true negatives compared to the other models, confirming its robustness and reliability for structural damage detection.

**Figure 13:**
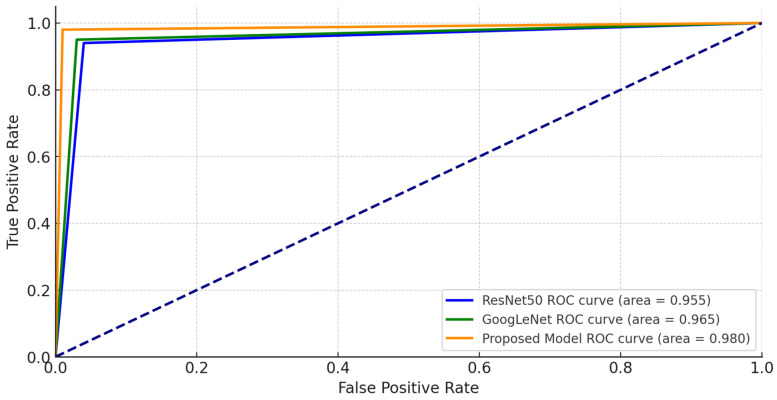
ROC curve of models.

We also employed receiver operating characteristic (ROC) curves to compare the classification effectiveness across models. Figure 13 illustrates the relationship between true positive and false positive rates, with our proposed model achieving an AUC-ROC of 0.980. This high AUC value underscores the model’s strong discrimination capability, effectively distinguishing damaged from undamaged structures across various thresholds. Although the proposed model required slightly higher computational resources and training time than ResNet50 and GoogLeNet, this additional cost is justified by the substantial gains in classification accuracy and reliability, making it highly suitable for real-world applications, particularly in scenarios like post-disaster assessments, where precision is crucial.

The accuracy curves for training and validation, displayed in Figure 11c, show a consistent improvement over epochs, with a close alignment between the curves by the end of training. This close alignment indicates that the model generalized well to unseen data and exhibited minimal overfitting, a notable advantage over the pre-trained models.

The proposed model required slightly more computational resources and longer training times compared to the pre-trained models. This could be a consideration in scenarios where computational efficiency is a priority.

## 6. Conclusions

The proposed system showed exceptional results in detecting structural damage. The ResNet50 model achieved an accuracy of 97.2%, with a precision of 97.1%, recall of 96.9%, and an F1-score of 97.0%. These figures demonstrate ResNet50’s strong capacity to detect damage, with a solid balance between precision and recall. Likewise, the GoogLeNet model delivered a slightly improved accuracy of 97.5%, with precision at 97.5%, recall at 97.3%, and an F1-score of 97.4%. This model’s ability to capture features across multiple scales proved beneficial for identifying structural damages. However, the proposed model outperformed both, achieving an accuracy of 98.6%, a precision of 98.2%, a recall of 98.8%, and an F1-score of 98.5%. The superior performance of our model can be credited to its advanced design, which excels at recognizing intricate patterns and subtle anomalies in data. This results in fewer false negatives, leading to more accurate detections, particularly in complex scenarios. Although these outcomes are promising, future work will focus on enhancing the model’s ability to handle more subtle and complex damage scenarios, with the aim of minimizing misclassifications and improving the overall accuracy, particularly in real-world environments where variations in conditions and structural differences may impact performance. Additionally, a key direction for future development involves integrating our AI model with real-time drone technology. This would enable drones to autonomously capture and analyze images of damaged structures on-site, providing immediate assessments of structural damage after disasters. Such a system would offer rapid, efficient, and accurate damage evaluation, supporting rescue and recovery teams by prioritizing areas needing immediate attention, thereby saving time, resources, and potentially lives.

## Data Availability

The original contributions presented in this study are included in the articlel. Further inquiries can be directed to the corresponding author(s).

## References

[1] Smith J., Doe J. (2022). Challenges in Post-Disaster Structural Damage Assessment. Int. J. Disaster Manag..

[2] Minaee S., Boykov Y., Porikli F., Plaza A., Kehtarnavaz N., Terzopoulos D. (2022). Image Segmentation Using Deep Learning: A Survey. IEEE Trans. Pattern Anal. Mach. Intell..

[3] Jiang Z., Li Y., Zhang Y. (2020). Deep Learning Techniques for Automated Structural Damage Detection in Buildings. J. Comput. Civ. Eng..

[4] Wu C., Wong K., Lam H. (2020). Automated Detection of Structural Damage in Buildings Using Convolutional Neural Networks. Eng. Struct..

[5] Lee H., Kim S. (2019). Crack Detection in Building Facades Using Convolutional Neural Networks. J. Struct. Eng..

[6] Yang L., Zhang X., Sun J. (2020). Deep Learning-Based Crack Detection in UAV Images for Infrastructure Inspection. Autom. Constr..

[7] Sun X., Hou C., Zhu L. Deep Learning-Based Automated Inspection of Concrete Structures Using UAV. Proceedings of the IEEE International Conference on Robotics and Automation (ICRA).

[8] Zhang Q., Yang J. (2021). Recent Advances in Image-Based Structural Damage Detection: A Review. J. Build. Eng..

[9] Yuqing Z., Khalid M. Structural Damage Recognition Using VGG16 Model. Proceedings of the International Conference on Image Processing.

[10] Cao W., Li Y., He Z. (2021). Crack Detection in Gusset Plate Welded Joints of Steel Bridges Using Deep Learning. J. Bridge Eng..

[11] Xiuying W., Li X. (2020). Concrete Crack Detection Using ResNet101-Based Image Segmentation. Autom. Constr..

[12] Zheng J., Wang H., Li J. Rail Surface Crack Detection Using YOLOv3 and RetinaNet. Proceedings of the IEEE Conference on Computer Vision and Pattern Recognition.

[13] Guo R., Zhou J. (2021). Pavement Crack Detection Using YOLOv5. IEEE Trans. Intell. Transp. Syst..

[14] Kong X., Li J. (2020). Vision-Based Metal Fatigue Crack Detection Using Video Feature Tracking. IEEE Trans. Ind. Electron..

[15] Wilson T., Diogo R. (2021). Deep Learning for Structural Damage Detection: A Case Study Using VGG16. Eng. Struct..

[16] Wan S., Guan S., Tang Y. (2023). Advancing Bridge Structural Health Monitoring: Insights into Knowledge-Driven and Data-Driven Approaches. J. Data Sci. Intell. Syst..

[17] Khan I.U., Jeong S., Sim S.H. (2024). Investigation of Issues in Data Anomaly Detection Using Deep-Learning- and Rule-Based Classifications for Long-Term Vibration Measurements. Appl. Sci..

[18] Mohamed A., El-Saadawi M., Sayed T. (2021). Disaster Resilience: Deep Learning Applications in Post-Earthquake Structural Assessment. Nat. Hazards Rev..

[19] Rathinam S., Madhavan P., Sivaramakrishnan C. (2020). Deep Learning for Post-Disaster Structural Damage Detection: A Review. J. Build. Pathol. Rehabil..

[20] Shabbir A., Ali N., Jameel A., Zafar B., Rasheed A., Sajid M., Ahmed A., Dar S. (2021). Satellite and Scene Image Classification Based on Transfer Learning and Fine Tuning of ResNet50. Math. Probl. Eng..

[21] Feng C., Zhang H., Wang S., Li Y., Wang H., Yan F. (2019). Structural damage detection using deep convolutional neural network and transfer learning. KSCE J. Civ. Eng..

[22] Lecun Y., Bottou L., Bengio Y., Haffner P. (1998). Gradient-Based Learning Applied to Document Recognition. Proc. IEEE.

[23] Nair V., Hinton G.E. Rectified Linear Units Improve Restricted Boltzmann Machines. Proceedings of the 27th International Conference on Machine Learning (ICML), Omnipress 2010.

[24] Huang G., Liu Z., van der Maaten L., Weinberger K.Q. Densely Connected Convolutional Networks. Proceedings of the IEEE Conference on Computer Vision and Pattern Recognition (CVPR).

[25] Krizhevsky A., Sutskever I., Hinton G.E. (2012). ImageNet classification with deep convolutional neural networks. Adv. Neural Inf. Process. Syst..

[26] He K., Zhang X., Ren S., Sun J. Deep residual learning for image recognition. Proceedings of the IEEE Conference on Computer Vision and Pattern Recognition.

[27] Zagoruyko S., Komodakis N. Wide Residual Networks. Proceedings of the British Machine Vision Conference (BMVC).

[28] Szegedy C., Liu W., Jia Y., Sermanet P., Reed S., Anguelov D., Erhan D., Vanhoucke V., Rabinovich A. Going deeper with convolutions. Proceedings of the IEEE Conference on Computer Vision and Pattern Recognition (CVPR).

[29] Szegedy C., Vanhoucke V., Ioffe S., Shlens J., Wojna Z. Rethinking the Inception Architecture for Computer Vision. Proceedings of the IEEE Conference on Computer Vision and Pattern Recognition (CVPR).

[30] Ioffe S., Szegedy C. Batch Normalization: Accelerating Deep Network Training by Reducing Internal Covariate Shift. Proceedings of the 32nd International Conference on Machine Learning (ICML).

[31] Woo S., Park J., Lee J.Y., Kweon I.S. CBAM: Convolutional Block Attention Module. Proceedings of the European Conference on Computer Vision (ECCV).

[32] Civera M., Fragonara L.Z., Surace C. (2019). Video Processing Techniques for the Contactless Investigation of Large Oscillations. J. Phys. Conf. Ser..

[33] Prechelt L. (1998). Early stopping-but when?. Neural Netw. Tricks Trade.

[34] Powers D.M. (2011). Evaluation: From precision, recall and F-measure to ROC, informedness, markedness and correlation. J. Mach. Learn. Technol..

